# Characteristics of Recurrent Atrial Arrhythmias and Lesion Assessment Late After Pulsed Field Ablation of Atrial Fibrillation

**DOI:** 10.1161/CIRCEP.125.014506

**Published:** 2026-09-18

**Authors:** Maxime Beneyto, Corentin Chaumont, Masaaki Yokoyama, Franck Raczka, Olivier Thomas, Nathan Marimpouy, Mickael Laredo, William Escande, Sandrine Venier, Gregoire Massoulié, Matthieu Granier, Gabriel Decebal Latcu, Pierre Ollitrault, Laure Champ-Rigot, Leslie Placide, Pascal Defaye, Antoine Lepillier, Estelle Gandjbakhch, Serge Boveda, Pierre Jaïs, Frederic Anselme, Frederic Sacher, Philippe Maury

**Affiliations:** 1 Toulouse University Hospital (M.B., N.M., P.M.).; 2 I2MC, INSERM UMR 1297, Toulouse (M.B., P.M.).; 3 Rouen University Hospital (C.C., F.A.).; 4 University Hospital Bordeaux, Pessac (M.Y., P.J., F.S.).; 5 IHU Liryc, Bordeaux University, INSERM 1045 (M.Y., P.J., F.S.).; 6 Clinique du Millénaire, Montpellier (F.R.).; 7 Clinique Ambroise Paré (O.T.).; 8 Clinique Pasteur 54918 , Toulouse (N.M., S.B.).; 9 University hospital La Pitié-Salpêtrière, Paris (M.L., E.G.).; 10 Centre Cardiologique du Nord 56509 , Saint-Denis (W.E., A.L.).; 11 University Hospital, Grenoble (S.V., P.D.).; 12 University Hospital, Clermont-Ferrand (G.M.).; 13 University Hospital Montpellier, France (M.G., L.P.).; 14 Centre Hospitalier Princesse Grace, Monaco (G.D.L.).; 15 University Hospital Caen, France (P.O., L.C.-R.).

**Keywords:** atrial fibrillation, atrial flutter, catheter, electroporation, fluoroscopy, pulmonary veins, pulsed field ablation

## Abstract

**BACKGROUND:**

Pulsed field ablation (PFA) is a novel and rapidly expanding energy for the ablation of atrial fibrillation (AF). The relationships between the characteristics of pulmonary vein (PV) and extra-PV PFA applications, and the type of arrhythmia recurrence or durable lesion observed during redo procedures remain unclear.

**METHODS:**

In this substudy of the FRANCE PFA national exhaustive multicentric registry, we collected detailed data on PFA applications delivered during the initial AF ablation procedure. Among patients with arrhythmia recurrence undergoing redo procedures with 3-dimensional mapping (n=145), we analyzed the type of recurrence, rates of durable PV isolation (PVI), and the presence of complete block over the ablation lines.

**RESULTS:**

Recurrent arrhythmias comprised AF in 58%, atrial tachycardia in 23%, and typical atrial flutter in 9%. AF was less frequently observed when initial ablation targeted extra-PV areas (45 versus 71%; *P*=0.01) and when a 35-mm diameter catheter was used for PVI only (50 versus 77%; *P*=0.01). Complete durable PVI was observed in 42% of patients (69% to 76% for each separate PV), and durable roof/PW and mitral isthmus blocks were observed in 62% and 31% of cases, respectively. The rate of durable PVI increased with the number of PFA applications (*P*<0.05), reaching 84% with >12 applications. Similarly, roof/PW block rates increased to 88% with >16 applications (*P*<0.01). Mitral isthmus block rate remained low (31%) and was not significantly associated with application number.

**CONCLUSIONS:**

In patients undergoing redo procedures after an initial AF ablation using the pentaspline PFA catheter, AF was less frequently observed when ablation lesions were performed beyond PVs. A high rate of durable PVI or block was achieved with >12 PFA applications per PV and >16 for the roof/PW. Long-term durability of mitral isthmus block was poor using the Pentaspline PFA catheter.

**WHAT IS KNOWN?**

- Pulsed field ablation may create pulmonary vein isolation or extravenous lesions, but the type of recurrence and factors of durable isolation or lesions remain unclear.

**WHAT THE STUDY ADDS**

- Recurring atrial arrythmias after pulsed field ablation for atrial fibrillation are diverse and somewhat related to the initial lesion set.
- A high durable isolation rate of the pulmonary veins or roof/posterior wall block may be achieved provided a sufficient number of applications are performed.
- The mitral isthmus appeared to be an unsuitable target for the pentaspline catheter.

Pulsed field ablation (PFA) has recently emerged as a novel energy source for atrial fibrillation (AF) ablation. It relies on repeated ultra-short high-voltage electrical pulses to generate nanoscale membrane pore formation leading to cardiomyocyte-preferential cell death.^1^

The pentaspline PFA catheter (Farawave, Boston Scientific, Marlborough, MA) was the first to be commercially released and is the most worldwide available device to date. It is a large-footprint (31 or 35 mm diameter), single-shot catheter designed for pulmonary vein isolation (PVI). Several studies have demonstrated its efficacy and safety^2–4^ with outcomes comparable to conventional thermal energies.^5–7^

Based on the first in-human trials, an optimized PFA delivery protocol was developed, consisting of 2 applications in basket conformation at the pulmonary vein (PV) antrum, followed by catheter rotation (36°) and 2 further applications, then repeating this sequence in flower configuration (8 applications in total).^8^ However, in routine clinical practice this protocol is frequently adapted, with increased numbers of applications, as reported in the FRANCE PFA registry.^9^ Additionally, off-label extra-PV applications are common (34% of patients).^9^

While durable PVI remains the cornerstone of AF ablation,^10^ recurrence rates after PFA at 1-year still range from 18% in paroxysmal to 28% in persistent AF in large series.^1^ In patients undergoing redo procedures, PV reconnections were observed in over half of cases.^11^ A descriptive analysis of recurrence mechanisms has recently been reported.^11^ However, the relationship between the parameters of initial PFA protocol (number of applications and catheter conformation) and the mechanisms of recurrence has not been thoroughly explored. In addition, the association between first-time procedural characteristics and lesion durability remains unknown.

In this substudy of the prospective and exhaustive FRANCE PFA registry, we aimed to (1) describe the mechanisms of recurrent atrial arrhythmias following initial AF ablation performed with PFA and (2) assess the relationships between initial procedural characteristics and both the mechanisms of recurrence and the durability of lesions observed at redo procedures.

## Methods

Data Availability Statement: data available on request from the authors.

### Study Design and Population

FRANCE-PFA is a nationwide multicenter registry that included all patients undergoing a first AF ablation using the Farawave catheter in France from March 2021 to February 2024.^9^ The registry prospectively collected detailed procedural data, including catheter size, number and location of PFA applications, and catheter conformation for each delivery. This substudy is an ancillary analysis of the 3306 patients enrolled in the FRANCE PFA registry up to September 2023. Some data are available on demand by contacting the corresponding author.

All patients with a documented relapse of AF, atrial flutter, or atrial tachycardia (AT) after the blanking period who underwent a redo procedure between 2021 and the end of 2023 were included. Patients were excluded if the redo procedure did not involve a 3-dimensional (3D) electroanatomic mapping of the left atrium (LA).

The lesion sets performed during the initial PFA procedure had been recorded (PV, roof/posterior wall [PW], mitral isthmus), together with the number of applications for each catheter conformation, and the acute results of PFA applications. PVI was defined as the lack of any detectable potential on the pentaspline catheter after the full set of applications (entry block) w/wo exit block. Additional roof/PW and mitral isthmus lesions (collectively referred to as PVI+) were assessed by the disappearance of all local potentials on the pentaspline catheter in the ablated region, w/wo additional mapping or pacing maneuvers to demonstrate linear block. Catheter contact was assessed on fluoroscopy only by visualizing the pentaspline deformation when pushed again the PV ostia or PW.

For redo procedures, medical records and 3D electroanatomic maps were retrospectively analyzed. When arrhythmias were present at baseline or inducible, the mechanisms were classified as AF, typical atrial flutter, or AT and further characterized. Furthermore, PVI durability, as well as roof/PW and mitral isthmus block, were assessed using 3D voltage and activation mapping during sinus rhythm or atrial pacing, as appropriate. Finally, scar areas at the PV antra (interrupted at the PV ostia, when the vessel is coming out of the LA shell), PW (between both right and left PV antrums), or mitral isthmus were measured on maps. We applied vendor-specific amplitude cutoffs to define dense scar (<0.5 mV for Abbott EnSiteX and Biosense Webster Carto 3, <0.2 mV for Boston Sc Rhythmia ) and system-specific software for measuring surfaces and LA volumes.

### Ethics

This study is an ancillary analysis from the FRANCE-PFA prospective nationwide registry (URL: https://www.clinicaltrials.gov; Unique identifier: NCT06497933)^9^ for which all participants gave written consent for the anonymous use of their routine clinical data for the purpose of research. Institutional review board approval was obtained from each participating center, and the registry was conducted in accordance with the Declaration of Helsinki.

### Statistical Analysis

We presented the results as number (percentage) or median (range) according to the type of variable. Comparisons between categorical variables were performed with Fisher exact tests. Proportions of isolated PVs and blocked PVI+ targets and their respective CIs were estimated with bootstrapping. The association between PFA delivery characteristics and PVI or PVI+ block was investigated using logistic regression. Mixed-effects models were employed to account for the shared variance of the PVs of a single individual and to obtain odds ratios (OR) with 95% CIs. The determinants of the extent of scar areas were investigated using linear regression. The following candidate explanatory variables: PFA catheter size, total number of applications per site, and catheter conformation were investigated.

All statistical tests relied on a threshold of *P*<0.05 and were performed with R version 4.4.0 (R Foundation for Statistical Computing, Vienna, Austria).

## Results

### Study Population

We included a total of 145 patients (33% females, n=48) with a median age of 67 (59–75). Most patients had no underlying cardiomyopathy (n=97, 67%) and displayed a preserved left ventricular ejection fraction (n=118, 83%). Baseline characteristics are summarized in Table 1.

**Table 1.**
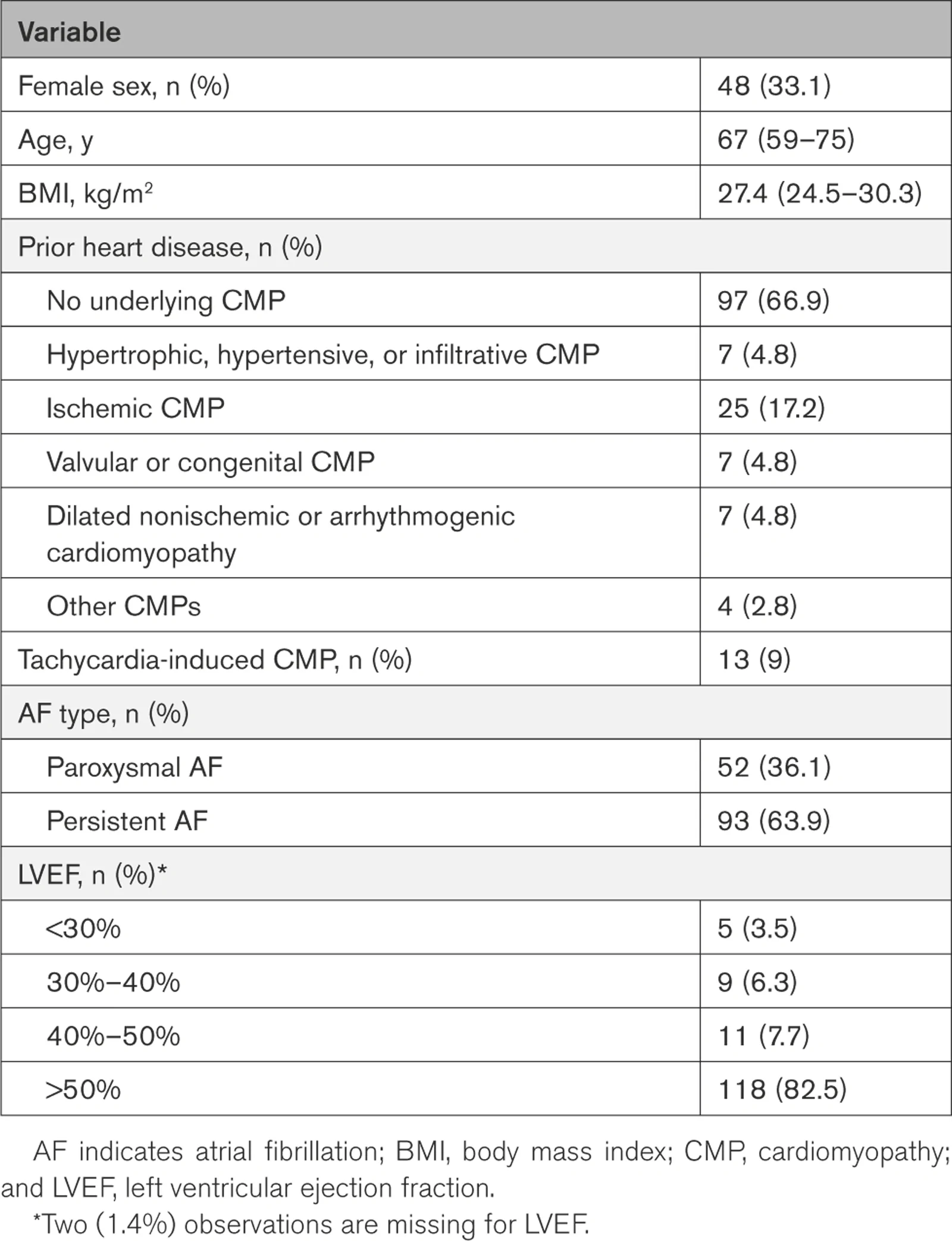
Patient Characteristics (n=145)

During the same period, 34 other patients from the France PFA registry experienced recurrent arrhythmias outside the blanking period, but were not included due to the absence of 3D mapping during the redo procedure (see flowchart in Supplemental Material).

### Index Procedure

The index ablation procedure primarily targeted persistent AF (n=96, 64%) and was guided by a 3D mapping system in only 10 cases (7%). The majority of procedures were performed using a 31-mm-diameter pentaspline PFA catheter (n=87, 60%). Half of the procedures targeted the PVs only (n=73, 50%). Only 57 (39%) patients received the recommended “4+4” applications per vein, while 11 patients (8%) received fewer applications and 77 (53%) received extra applications. The median number of applications for all 4 PVs was 16 baskets + 16 flowers (ranging from 16+16 to 20+24, respectively). Further details of applications per PV are provided in Supplemental Material. When adding the PVI+ applications, the median number of applications per procedure was 41 (32–60).

PVI+ applications consisted of roof/PW lesions in 37 patients (51%), mitral isthmus lesions in 2 (3%), and both in 34 (47%). The median number of applications was 14 (9–20) for the roof/PW and 12 (9 to 16) for the mitral isthmus. Most PVI+ applications were performed in a flower conformation at the roof/PW (n=49, 69%) as well as at the mitral isthmus (n=34, 94%).

PV exit block was systematically checked in 7 centers (representing 57 patients with redo procedures). At the end of the initial procedure, complete PVI was achieved in all cases, as well as roof/PW and mitral isthmus block, when checked.

The median procedure duration was 60 minutes (46–80), and the median fluoroscopy duration was 17 minutes (13–25).

### Redo Procedure

Atrial arrhythmias recurred after a median interval of 5 months (3–8), and the redo procedure with a 3D mapping system was performed 7 months (5–10) after the index procedure. The 3D mapping systems were Biosense Carto 3 in 83 patients (57%), Boston Sc Rhythmia in 60 (41%), and Abbott EnSiteX in 2 (1%).

Recurrence comprised AF in 84 (58%), AT in 33 (23%), and typical atrial flutter in 13 (9%), while precise mechanisms remained undetermined in 15 (10%; Table 2).

**Table 2.**
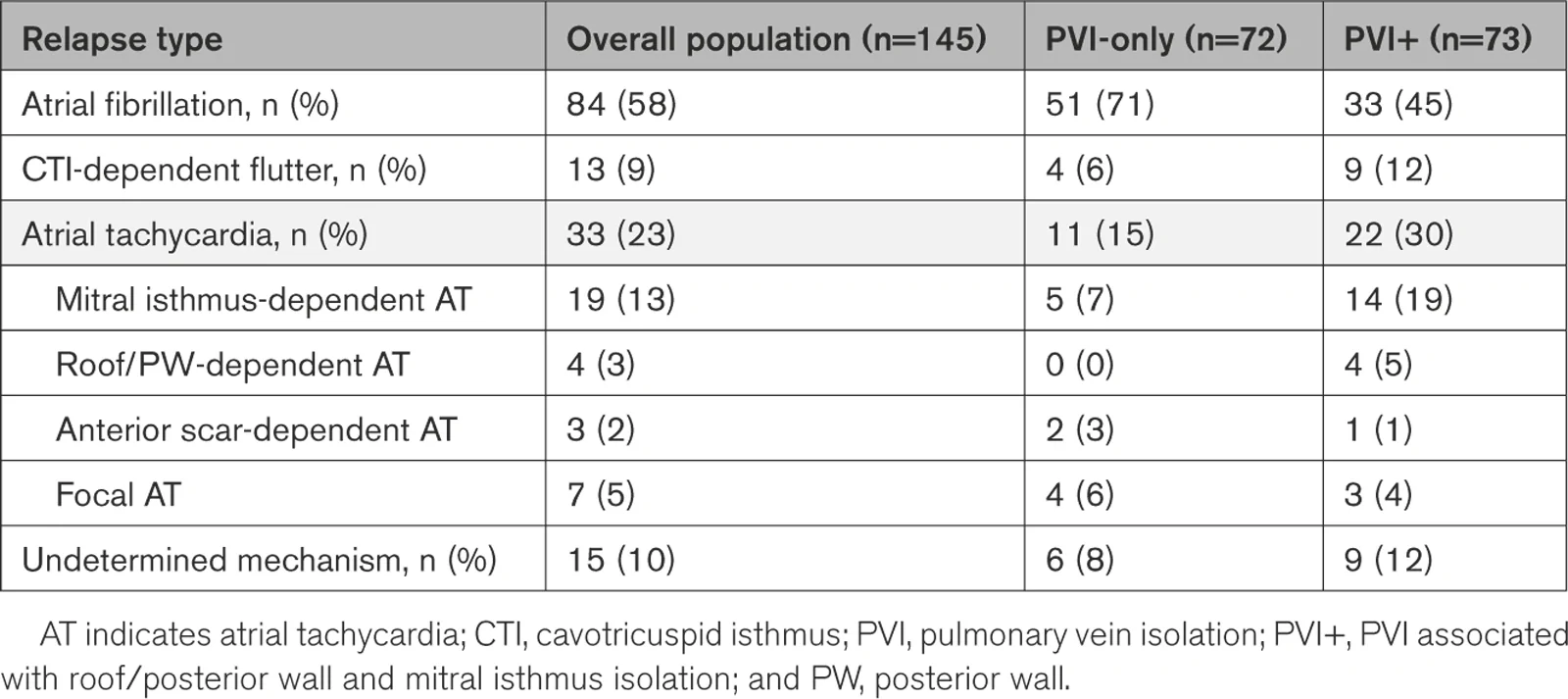
Types of Recurring Atrial Arrhythmias

Type of recurring arrhythmia significantly differed according to the initial lesion set: AF recurrence was significantly less frequent when the initial ablation extended beyond the PVs (45 versus 71%; *P*=0.01), even after taking the initial type of AF into account (*P*=0.01). Mitral isthmus-dependent AT was observed in 11 (31%) patients having undergone mitral isthmus applications, while roof-dependent AT was noted in only 4 (5%) patients with previous roof/PW applications. In PVI-only patients, 5/72 (7%) cases of mitral isthmus-dependent AT were observed, but no case of roof-dependent AT.

There were no significant differences in scar surfaces at the end of the index procedure (in the 6 cases where 3D mapping has been used and in whom maps were available) compared with what was observed at the redo procedure (Supplemental Material).

In PVI-only patients, the catheter size was associated with a change in the type of relapse (*P*<0.01), shifting from an AF-dominant pattern (77%) when the 31 mm diameter catheter was used during the index procedure, to a more balanced 1 (50%) with AT almost as much represented as AF when PVI was achieved using the 35 mm (Figure 1; Supplemental Material). This was not the case in PVI+ patients, with around 44% of recurrent AF regardless of the catheter size (*P*=0.92).

**Figure 1.**
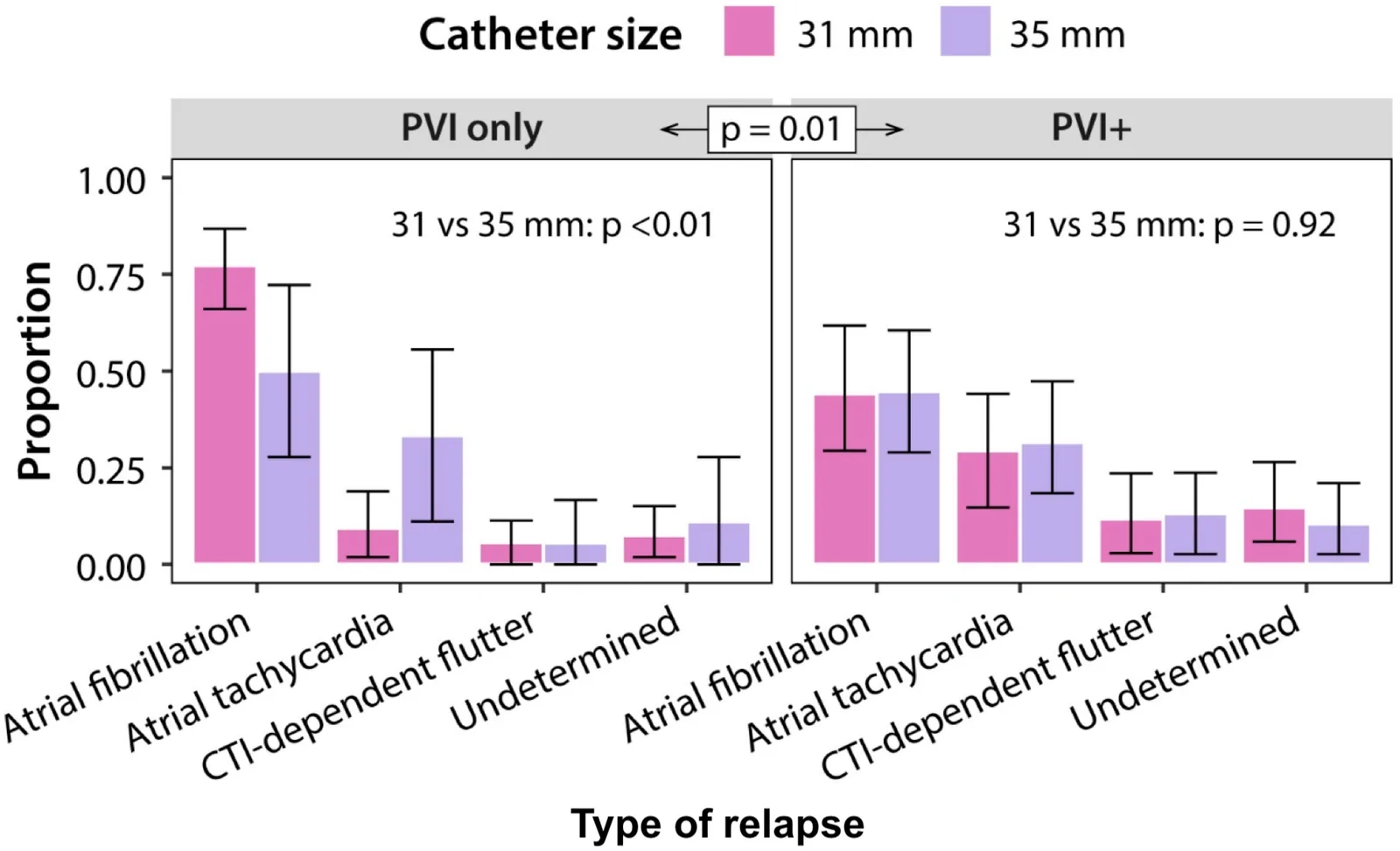
**Distribution of type of relapse according to initial ablation strategy and catheter size (see text for details).** CTI indicates cavo-tricuspid isthmus; PVI, pulmonary vein isolation; and PVI+, pulmonary vein isolation plus extra-pulmonary vein lesions.

At redo procedures, all PVs were found isolated in 60 patients (42%; 40 patients with 1 PV reconnection, 28, 8, and 9 patients with 2, 3, and 4 PV reconnections, respectively). There was no significant difference in reconnection rates among PVs, with the most frequently isolated being the left inferior (n=100, 76%), followed by the left superior (n=105, 73%), right inferior (n=98, 71%), and right superior (n=100, 69%; *P*=0.63). The median PV scar area was 28 cm^2^ (17–50).

Among 71 patients with roof/PW targeted during the index procedure, durable block was observed in 44 (62%) at redo procedures. Roof/PW scar area displayed a 13 (8 to 20) cm^2^ extent. In 3/74 (4%) patients with an ablation strategy not including the roof/PW, the roof/PW was found completely blocked. In these few patients, the LA size ranged from 103 to 228 cm^3^ (normal values <35 cm^3^/m^2^), the PVs received a total of 32 to 41 applications, and a 31-mm catheter was used in 2/3 cases.

Among the 36 patients with mitral isthmus targeted at the index procedure, complete block was observed in 11 (31%) of redo procedures. The mitral isthmus area displayed a 4 (2 to 10) cm^2^ scar extent. In 5/109 (7.5%) patients with an ablation strategy not including the mitral isthmus, the mitral isthmus was also found completely blocked. In these few patients, the LA size ranged from 103 to 402 cm^3^ (median length between LIPV and mitral isthmus 31.5 mm, from 27–45 mm), the PVs received a total of 32 to 56 applications, and a 31-mm catheter was used in 4/5 cases.

### Mechanisms of Recurring Arrhythmias

Outside AF and CTI-dependent atrial flutter, recurring arrhythmias (n=48) investigated at the redo procedure were AT (roof-dependent reentry n=4, perimitral reentry n=19, anterior left atrial reentry n=3 and focal AT n=7), or were of undetermined mechanism (n=15; Table 2).

### Correlations Between Lesions and Type of Recurrence

AF was the recurring arrhythmia in 50% (n=30) of patients with complete PVI at redo procedure versus 64% (n=54) of patients with at least 1 reconnected PV (*P*=0.07). In patients with recurring AT, 27 (54%) displayed complete PVI.

### Factors Associated With Durable PVI and Extent of PV Scar

Analysis included 560 PVs and 2539 basket applications, and 2959 flower applications.

Use of a 35 mm versus 31 mm catheter did not significantly modify durable PVI (74% versus 71%; OR, 1.30 [0.68–2.49]; *P*=0.43). PVI rate increased with the number of PFA applications up to a plateau (13–20 applications), then dropped abruptly (Figure 2; Supplemental Material). This pattern was consistent across all 4 PVs (Supplemental Material). The optimal number of applications by PV seemed to be 13 to 16, yielding an 84% (80–88) probability of durable isolation among patients with recurring arrythmias undergoing a redo-procedure.

**Figure 2.**
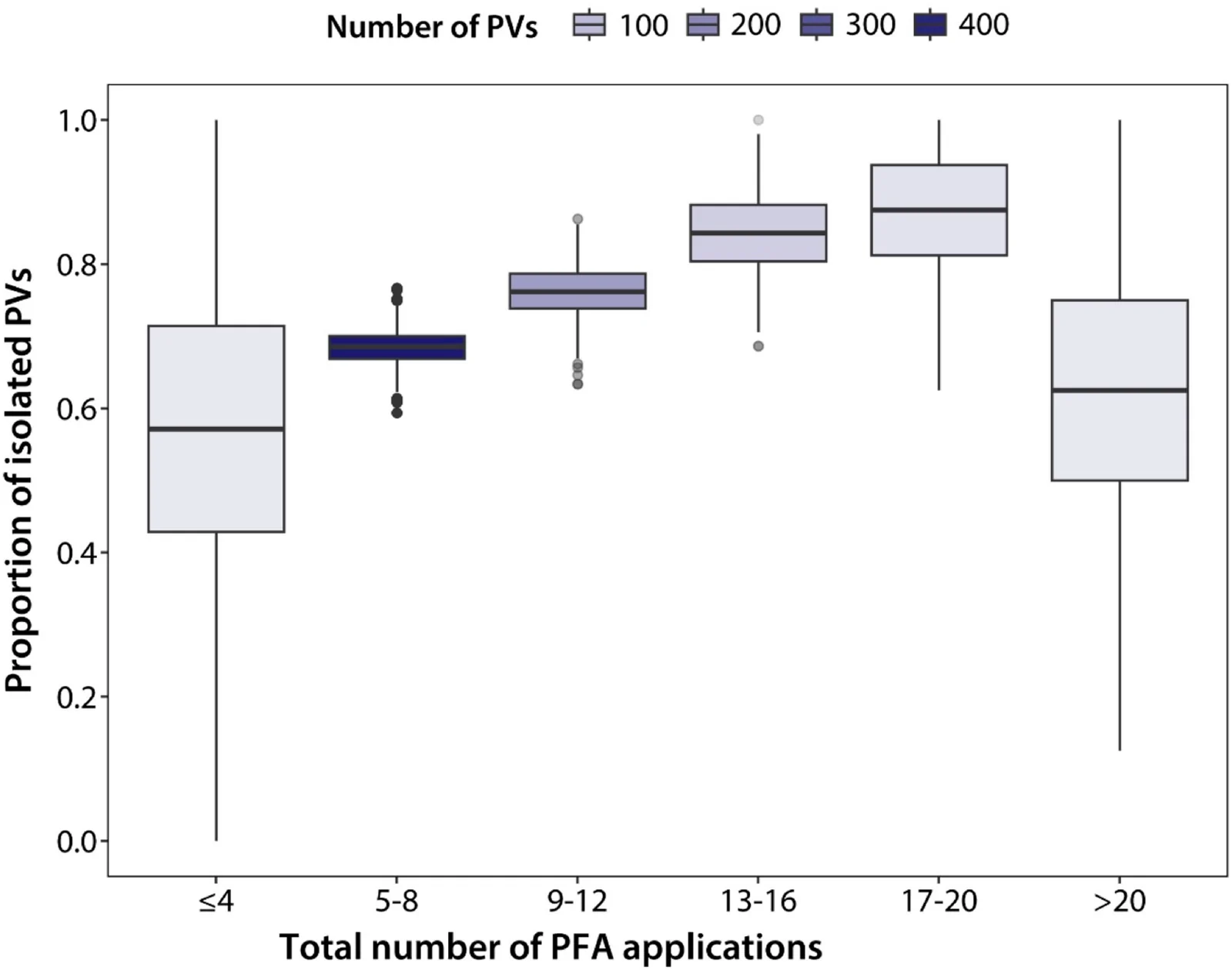
**Proportion of isolated pulmonary veins (PVs) according to the number of pulsed field ablation (PFA) applications (*P*<0.05, see text).** Pulmonary vein isolation rate increased with the number of PFA applications. Exact values and CIs are available in the Supplemental Material.

The drop in PVI probability concerned 8 PVs (1.4%) that received >20 applications. We considered these few PVs outliers, particularly difficult to isolate for electrophysiological or anatomic reasons. Therefore, they were excluded from subsequent analysis. In the remaining PVs (n=552), the number of applications was significantly associated with durable PVI (OR, 1.13 [1.02–1.24]; *P*=0.02), even after multivariate adjustment for sex, age, body mass index, and existence of any cardiomyopathy (OR, 1.13 [1.03–1.24]; *P*=0.02). Left atrial volume could not be added to the multivariate model due to heterogeneity in imaging before the index procedure, but recent datas tend to prove that anatomy is marginally associated with the efficiency of PFA.^12^

Number of basket applications and flower applications alone were both associated with a nonsignificant trend towards a higher success rate of PVI (OR, 1.18 [0.99–1.41]; *P*=0.07 and 1.12 [0.99–1.27]; *P*=0.06, respectively). The extent of PV scar was not different when a 35-mm catheter was used instead of a 31-mm catheter (*P*=0.89) and was not related to the number of applications (*P*=0.87).

### Factors Associated With Durable PVI+ and Extent of PVI+ Scar

#### Roof/PW

The analysis investigated 71 roofs/PWs and a total of 180 basket applications and 991 flower applications.

The use of a 35-mm compared with a 31-mm catheter did not increase the probability of durable roof/PW block (59% versus 64%; OR, 0.84 [0.32–2.20]; *P*=0.72). Higher rates of roof/PW block were observed using flower versus basket conformation: 78% versus 29%; OR, 8.63 (2.71–27.56), *P*<0.001. The rate of long-term roof/PW block was significantly related to the number of PFA applications (OR, 1.12 [1.04–1.21]; *P*<0.01; Figure 3). The more applications on the roof/PW, the more likely it was found blocked at redo procedures (OR, 1.12 [1.04–1.21]; *P*<0.01). Delivering >16 applications yielded an 88% (80–94) chances to block the roof/PW. The extent of roof/PW scar after PFA was related to both the catheter diameter (*P*<0.001) and the number of applications (*P*<0.0001).

**Figure 3.**
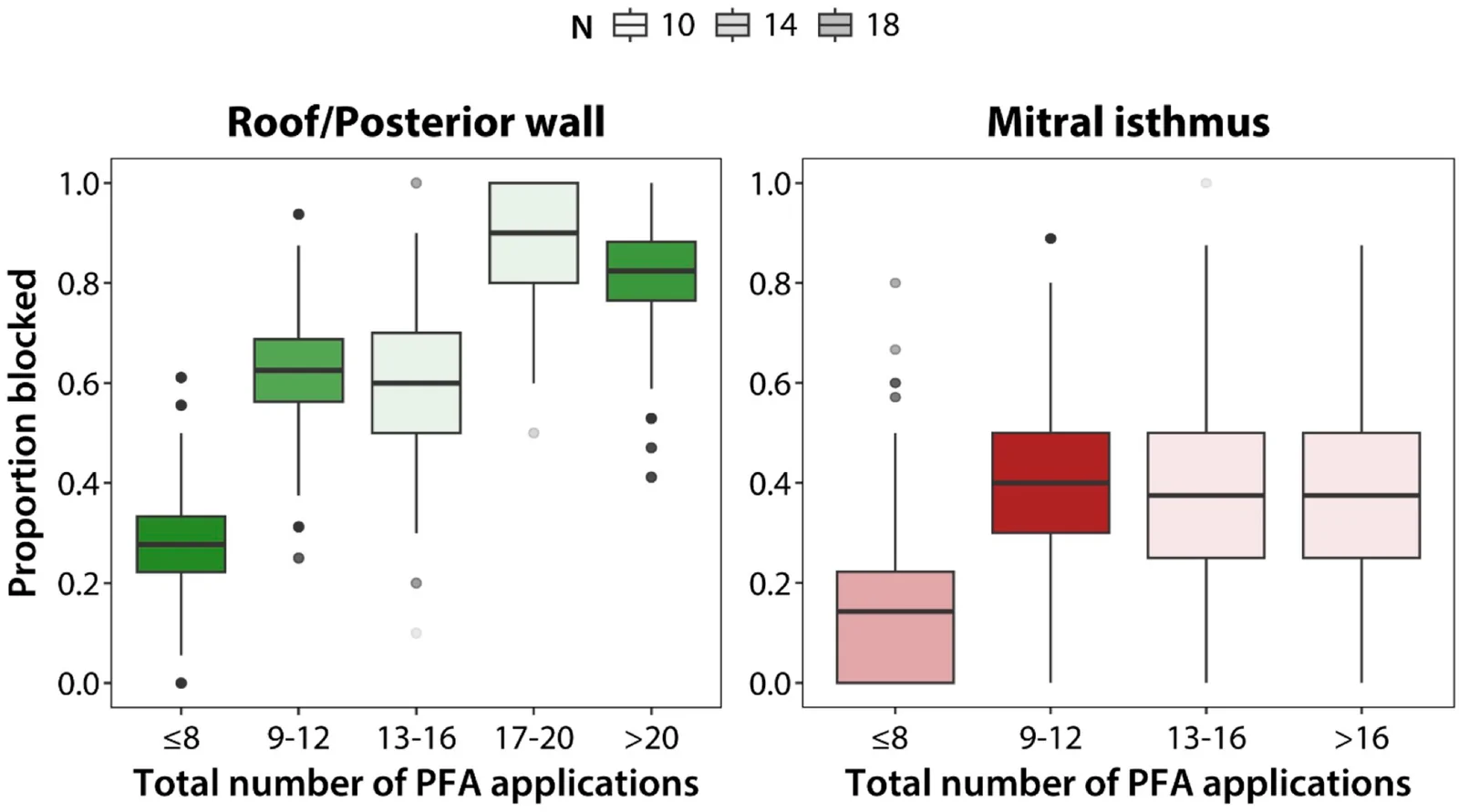
**Proportion of blocked roof/posterior wall (PW; *P*<0.05) and mitral isthmus (*P*=ns) according to the number of pulsed field ablation (PFA) applications (see text).** The rate of long-term roof/PW block was significantly related to the number of PFA applications, while the proportion of long-term mitral isthmus block remained low and not correlated to the number of applications.

#### Mitral Isthmus

The analysis investigated 36 mitral isthmuses and a total of 18 basket applications and 522 flower applications.

The use of a 35-mm catheter was not correlated with the probability of durable mitral isthmus block (13% versus 21%; OR, 0.31 [0.07–1.47]; *P*=0.14). Only 2/36 (6%) mitral isthmuses were ablated using the PFA catheter in a basket conformation. Thus, the efficacy of basket versus flower conformation cannot be compared. The number of applications was not associated with the proportion of long-term mitral isthmus block (OR, 1.03 [0.98–1.10]; *P*=0.26), which reached only 31% (17–47; Figure 3). The extent of mitral isthmus scar after PFA was larger with a 35-mm catheter (*P*=0.04) and when more applications were delivered (*P*<0.01).

Additional comparisons between scar areas, LA volumes and normalized scar areas and catheter size, number of applications, presence of conduction block and type of recurrences are given in Supplemental Material. Grossly, there was no relationship between scar areas and the type of relapse (except for larger PW scar surfaces in patients with AT versus AFib); PW and mitral scars and LA volume were larger in cases of the use of a 35 mm diameter catheter and positively correlated to the number of applications and to conduction block.

## Discussion

In this study including all patients with recurring atrial arrhythmias investigated with 3D mapping systems after a first AF ablation using pulse fields delivered by the Boston Scientific/ Farapulse system, the main findings were (1) delivering more than 4+4 PFA applications is associated with a higher probability of durable PVI, culminating at almost 85% when 13 to 20 applications were delivered on each PV, (2) PFA is effective for creating roof/PW lesions and the probability of block is dose-dependent, culminating at around 90% when >16 applications were delivered, (3) the efficacy of PFA for mitral isthmus isolation is poor whatever the number of applications delivered, and (4) although no specific type of recurring arrhythmia seemed associated with the initial PFA applications, less AF and more ATs recurred when a PVI+ strategy was performed, whatever the type of AF, and less AF and more ATs were observed using a 35 mm diameter catheter in PVI only patients.

### General Considerations

As this study included patients with recurring arrhythmias, persistent AF at the index procedure was much more common than in the MANIFEST-PF registry (64% versus 32%).^1^ Similarly, a PVI+ strategy was much more common (40% versus 23%). The time to relapse and the time to redo were rather short. The higher prevalence of PVI+ strategy may explain the lower proportion of AF as the relapse rhythm than in MANIFEST-REDO or EU-PORIA (58% versus 81% and 66%, respectively).^11,13^ However, the proportion of redo procedures revealing a durable isolation of all PVs was similar (42% versus 45% and 36%).

The precise mechanisms of the recurring ATs were also reported in the EU-PORIA registry. Compared with our results, roof/PW-dependent ATs were much more common (28% versus 8%). Other mechanisms were roughly equally distributed. Again, this difference may be explained by the very limited use of a PVI+ strategy in EU-PORIA (16%).^13^

### PFA for PVI

For the first time, we were able to investigate the relationship between the number of PFA applications and (1) the extent of scar generated by the ablation and (2) the rate of durable PVI. We observed that a higher number of applications was not associated with larger PV scars. We hypothesize that, due to the conic shape of the PV antrum and the guidance of the support wire, the pentaspline PFA catheter ought to fall into roughly the same area around the PV ostium. Therefore, applications tend to stack, and the extent of the scar does not increase.

Conversely, we showed that going beyond the 4+4 protocol was associated with improved long-term PVI, reaching more than 80%, which is higher than the rate of PVI observed using other energies (65%–73%)^6^ in patients with recurring arrhythmias. This rate of long-term PVI is likely even higher in patients without recurrences. These results suggest that repeating PFA applications may be sufficient for achieving a very high rate of PVI, possibly allowing less attention to positioning and contact of the device, and thus probably obviating the need for 3D mapping for assessing PVI in the vast majority of patients. Moreover, recent data from France PFA registry tend to prove that PV anatomy is only marginally associated with the efficiency of PFA.^12^ Comparative studies with 3D mapping are needed, but in an intention to render procedures simpler, rapid, straightforward, and reproducible, we feel that simply repeating applications is probably superior to any other strategy.

A plateau was observed over 13 to 20 applications, which is probably the optimal range of applications to deliver on the PVs. Delivering more applications - the only relevant parameter that can be acted on by the operator—has been shown to create deeper and more effective lesions.^14^ Additionally, the more applications, the more likely the catheter is positioned on a residual gap with good contact. Recently, a simpler protocol consisting of 2+2 applications rather than 4+4 has been evaluated,^15^ with both strategies showing similar acute outcomes. Our data suggest that 4+4 does not give the patient the highest chances of PVI and it may therefore be the same or even worse with 2+2 applications.

Beyond 20 applications, a dramatic drop in PVI rate was seen in our population, but it concerned only very few cases. The most reasonable explanation for this phenomenon is that this limited number of PVs that required so many applications probably represent either tortuous anatomies, impeding the proper application of the pentaspline PFA catheter, or atrial tissue unusually resistant to PFA.

A trend between more applications and higher rates of durable PVI was observed with both basket and flower conformations, but due to the substantial correlation between the number of applications in each conformation, resulting in a lack of statistical power, it was not possible to determine which contributed the most to PVI in a multivariate analysis.

Finally, using a 35-mm diameter catheter did not result in larger scars or a higher rate of durable PVI. The expected larger field of PF delivery may have been counteracted by difficulties in maneuvering a larger catheter and positioning it with good contact at some PV antra. Moreover, in PVI-only patients, despite lesions of similar extent, we observed a higher occurrence of AT as the relapse arrhythmia when a 35-mm catheter was used. Such a catheter, when employed in a nondilated atrium, likely encroaches on the roof/PW or on the mitral isthmus, potentially leaving a narrow channel of viable tissue with reduced conduction velocity, promoting the onset of roof- or mitral-dependent flutter. Only a few patients with a PVI-only procedure ended up with a blocked roof/PW or mitral isthmus, while we observed 5 mitral-dependent ATs and not a single case of roof-dependent AT in this subset of our population. A PVI-only strategy does not seem to promote a relevant risk of such pro-arrhythmic consequences, and despite the safety profile of PFA, it appears unjustified to adopt a systematic PVI+ strategy as a counteracting measure.

### PFA for PVI+

The MANIFEST-PF registry has shown that a PVI+ strategy did not reduce the rate of recurrences after PFA.^1^ However, MANIFEST-REDO showed discreetly better outcomes in persistent AF patients who underwent roof/PW isolation despite the fact that only 46% of targeted roof/PWs were blocked.^11^ Noteworthily, the number of applications and catheter conformation for roof/PW PFA in both MANIFEST registries were not mentioned because such data were simply not available, in opposition to our work. We believe that our results may reignite the interest in roof/PW isolation as we give clues to promote more efficacious lesions which may in turn improve outcomes after AF ablation.

We were also able to investigate the procedural factors associated with the durability and extent of PVI+ lesions. For both roof/PW and mitral isthmus PFA, the more applications, the larger the scars observed during the redo procedure. Larger scar areas were also observed when a 35-mm catheter was used. However, this apparent increased efficacy in lesion formation was not systematically associated with an increased probability of block.

The overall proportion of blocked roof/PW was 62% but depended on the number of applications. Delivering >16 applications allowed an almost 90% rate of roof/PW block, a proportion similar to what Yokoyama et al^16^ reported in their recent single-center study (81%). Similar to PVI, durable roof/PW block is achievable with a high success rate when delivering a large enough number of applications. Again, it suggests that the need for 3D mapping is probably superfluous in most patients, and additional studies are needed for assessing the true role of 3D mapping for roof/PWI. Finally, roof/PW block was more frequently observed when PFA applications were delivered in a flower conformation rather than a basket conformation. We hypothesize that the electrode-to-atrial tissue contact was better in the former than the latter, promoting lesion durability and transmurality, or because of slightly larger lesions with the flower conformation.

The mitral isthmus proved to be rather resistant to PFA since only 31% were found blocked at redo procedures despite apparent complete block at the initial procedure. This finding is in line with the data published by Yokoyama et al^16^ reporting a complete lack of efficacy, mostly due to deep/epicardial connections. Previously, however, Davong et al^17^ had shown 100% acute mitral isthmus block with only 20% reconnections at 3 months. These results may be explained by (1) a transient stunning effect of PFA, obscuring the possibility of any reliable assessment of the block, or (2) a low use of 3D mapping to evaluate the block, which may have missed discrete residual gaps, and (3) a limited follow-up duration. Anatomically, the mitral isthmus is thick and multilayered with endocardium, fat infiltration, and coronary sinus/great cardiac vein epicardial musculature.^18^ Fat is a good thermal but weak electrical insulator, but it creates dramatic distortions in the electric field generated by PFA,^19^ making in vivo lesions hard to predict. Furthermore, the pentaspline PFA catheter is not able to deliver PFA on the epicardial side of the mitral isthmus through coronary sinus access. For these reasons, the mitral isthmus appears refractory to PFA, at least using this device. In our study, neither the number of applications nor the size of the catheter affected the probability of mitral isthmus block.

### Strengths and Limitations

The strengths of this study are its nationwide multicentric design comprising high-volume centers and its prospective and exhaustive collection of procedural characteristics concerning the delivery of PFA. This allowed us to investigate the association between PFA applications, mechanisms of recurrent arrhythmias, and lesion durability, which had not been undertaken hitherto.

The main limitation of our study is the observational and retrospective design. Despite our efforts to standardize scar measurements, there may remain some heterogeneity across centers, due to different 3D mapping systems and variations in LA anatomy. We limited our analysis to the most common PFA targets despite growing trends to perform cavotricuspid isthmus or superior vena cava isolation with PFA; for example, Catheter contact could only be assessed on fluoroscopy, thus newer techniques measuring tissue contact could have optimized our results,^20^ even if this remains to be demonstrated and if the importance of contact force in PFA is still unclear.^21^

Finally, our study was not designed to investigate complications of PFA. Increasing the number of PFA applications may entail the risk of side effects such as hemolysis. The number of applications preconized here is indeed close to that determined to be at risk of severe hemolysis.^22^ However, reported cases of clinically significant hemolysis are exceptional,^23^ even with up to 16 applications per vein.^24^ Another point of concern is that a high number of applications may also promote tissue heating.^25^ However, it is unlikely to generate thermal lesions as the minimum delay between applications (fixed by the manufacturer) is long enough to let the tissue cool down.^14^ Although never reported, dedicated investigations may be helpful to assess the potential side effects of multiple PFA applications.

### Conclusions

Recurring atrial arrhythmias after PFA for AF are diverse and somewhat related to the initial lesion set. In patients investigated after recurrence of atrial arrhythmias, durable PVI may be achieved in >85% when 13 to 20 applications by PV were performed, and durable roof/PW block in >90% for >16 applications. In our opinion, increasing the number of applications may raise interrogations about the need for 3D mapping for PVI or roof/PW ablation. The mitral isthmus appeared to be an unsuitable target for the pentaspline PFA catheter. Catheter size was not linked to the rate of durable lesions, and flower conformation seemed more efficient for durable PVI+ lesions.

## ARTICLE INFORMATION

### Disclosures

Dr Anselme is a consultant for and has received lecture fees from Boston Scientific, Medtronic, and Microport cardiac rhythm management (CRM). Dr Gandjbakhch has received honoraria from Boston Scientific, Medtronic, Microport CRM, Abbott, and Biotronik. Dr Jaïs is a consultant for and has received lecture fees from Boston Scientific, Biosense Webster, Afferra, and Stock from InHeart. Dr Massoulié received consulting fees from Boston Scientific. Dr Ollitrault received consulting fees from Abbott, Biotronik, Boston Scientific, Medtronic, Microport, and Zoll. Dr Sacher received modest consulting fees and speaking honorarium from Abbott, Biosense Webster, Boston Scientific, Inheart and Medtronic. The other authors report no conflicts.

### Supplemental Material

Tables S1–S8

Figures S1–S2

## Supplementary Material
