# Supplementary material for: Characteristics of Recurrent Atrial Arrhythmias and Lesion Assessment Late After Pulsed Field Ablation of Atrial Fibrillation
Source: Circ Arrhythm Electrophysiol. 2026 Sep 18;19(10):e014506. doi: 10.1161/CIRCEP.125.014506 (PMC13686636; doi:10.1161/CIRCEP.125.014506)
Supplement: Supplementary file 1 [file hae-19-e014506-s001.pdf]

Supplemental material

Supplemental Table 1

Distribution of recurring arrhythmias (in proportions) according to catheter size and ablation strategy

Relapse rhythm	31-mm pentaspline PFA catheter		35-mm pentaspline PFA catheter	
	**PVI-only (N=53)**	**PVI+ (N=34)**	**PVI only (N=18)**	**PVI+ (N=38)**
Atrial fibrillation	0.770 (0.660 – 0.890)	0.440 (0.270 – 0.620)	0.500 (0.278 – 0.722)	0.447 (0.289 – 0.605)
Focal atrial tachycardia	0.151 (0.057 – 0.245)	0.118 (0.029 – 0.235)	0.056 (0.000 – 0.167)	0.026 (0.000 – 0.079)
CTI–dependent flutter	0.038 (0.000 – 0.094)	0.088 (0.000 – 0.206)	0.056 (0.000 – 0.167)	0.132 (0.026 – 0.237)
Left atrial reentry	0.019 (0.000 – 0.057)	0.235 (0.118 – 0.382)	0.333 (0.111 – 0.556)	0.289 (0.158 – 0.421)
Other	0.019 (0.000 – 0.057)	0.118 (0.029 – 0.235)	0.056 (0.000 – 0.167)	0.105 (0.026 – 0.211)

Supplemental Table 2

Number of PFA applications according to pulmonary vein and catheter conformation (median, range)

Pulmonary vein	Basket applications	Flower applications	Total applications
LSPV	4 (4 – 6)	4 (4 – 6)	8 (8 – 12)
LIPV	4 (4 – 4)	4 (4 – 4)	8 (8 – 8)
RSPV	4 (4 – 6)	4 (4 – 6)	8 (8 – 12)
RIPV	4 (4 – 4)	4 (4 – 6)	8 (8 – 10)

Supplemental Table 3

Proportion of durable PVs according to the number of PFA applications

Number of applications	N	Proportion of isolated PVs (95% bootstrapped CI)
≤4	7	0.57 (0.43 – 0.71)
5 – 8	347	0.69 (0.67 – 0.70)
9 – 12	131	0.76 (0.74 – 0.79)
13 – 16	51	0.84 (0.80 – 0.88)
17 – 20	16	0.88 (0.81 – 0.94)
>20	8	0.63 (0.50 – 0.75)

Supplemental Table 4

Scar surfaces, LA volume and type de recurrence

	Atrial fibrillation	Atrial tachycardia	p =
Left atrial volume (cm^2)	176 ± 61	185 ± 54	0.41
Total scar surface (cm^2)	55 ± 47	53 ± 33	0.82
Total scar surface (%)	33 ± 23	29 ± 16	0.24
Pulmonary vein scar surface (cm^2)	35 ± 22	36 ± 29	0.84
Pulmonary vein scar surface (%)	21 ± 15	19 ± 13	0.5
Posterior wall scar surface (cm^2)	7 ± 9	13 ± 11	0.0008
Posterior wall scar surface (%)	5 ± 5	± 5	0.04
Mitral scar surface (cm^2)	4.4 ± 8	3.8 ± 5	0.63
Mitral scar surface (%)	6 ± 1	6 ± 7	0.96

Supplemental Table 5

Scar surfaces, LA volume and catheter diameter

	31 mm	35 mm	p =
Left atrial volume (cm^2)	170 ± 54	193 ± 60	0.03
Total scar surface (cm^2)	47 ± 30	62 ± 51	0.05
Total scar surface (%)	31 ± 18	31 ± 21	0.99
Pulmonary vein scar surface (cm^2)	36 ± 25	35 ± 25	0.88
Pulmonary vein scar surface (%)	21 ± 16	19 ± 13	0.31
Posterior wall scar surface (cm^2)	7 ± 9	14 ± 10	0.0006
Posterior wall scar surface (%)	6 ± 6	7 ± 5	0.11
Mitral scar surface (cm^2)	3 ± 5	5.5 ± 8	0.04
Mitral scar surface (%)	5 ± 8	8 ± 8	0.06

Supplemental Table 6

Scar surfaces, LA volume and number of PFA applications

	z =	p =
Nb applications PV vs surface scar PV	-1.8	0.85
Nb applications PV vs surface scar PV/LA volume	-0.6	0.55
Nb applications PV vs LA volume	0.3	0.77
Nb applications PW vs surface scar PW	6	<0.0001
Nb applications PW vs surface scar PW/LA volume	6.6	<0.0001
Nb applications PW vs LA volume	0.7	0.4
Nb applications mitral vs surface scar mitral	2.5	0.01
Nb applications mitral vs surface scar mitral/LA volume	1.9	0.05
Nb applications mitral vs LA volume	1.5	0.11

	z =	p =
Nb applications « basket » PV vs surface scar PV	1	0.32
Nb applications « basket » PV vs surface scar PV/LA volume	0.6	0.54
Nb applications « basket » PV vs LA volume	0.45	0.65
Nb applications « flower » PV vs surface scar PV	-0.8	0.41
Nb applications « flower » PV vs surface scar PV/LA volume	-1.2	0.23
Nb applications « flower » PV vs LA volume	0.15	0.88

Supplemental Table 7

Scar surfaces, LA volume and PVI or linear block

	No PV isolation	PV isolated	p =
Left atrial volume (cm^2)	176 ± 58	187 ± 57	0.31
Pulmonary vein scar surface (cm^2)	34 ± 26	38 ± 25	0.36
Pulmonary vein scar surface (%)	19 ± 15	31 ± 13	0.35
	No PW block	PW blocked	
Left atrial volume (cm^2)	180 ± 60	182 ± 60	0.87
Posterior wall scar surface (cm^2)	5 ± 6	18 ± 10	< 0.0001
Posterior wall scar surface (%)	3 ± 3	10 ± 5	< 0.0001
	No mitral block	Mitral block	
Left atrial volume (cm^2)	180 ± 54	189 ± 81	0.58
Mitral scar surface (cm^2)	3.3 ± 6.3	9 ± 8	0.001
Mitral scar surface (%)	5 ± 9	10 ± 8	0.03

Supplemental Table 8

Comparisons between scar surfaces

at the first and redo procedures (6 patients)

	Median (range)	p = (Wilcoxon test)
Pulmonary veins scar surface (cm^2)	38 (18-54) initial<br>54 (33-95) redo	0.11
Posterior wall scar surface (cm^2)	18 (10-27) initial<br>13 (7-18) redo	0.22
Mitral isthmus scar surface (cm^2)	7 (6-8) initial<br>6 (4-12) redo	1
Total scar surface (cm^2)	53 (40-68) initial<br>66 (46-117) redo	0.34

Supplemental Figure 1

Flowchart

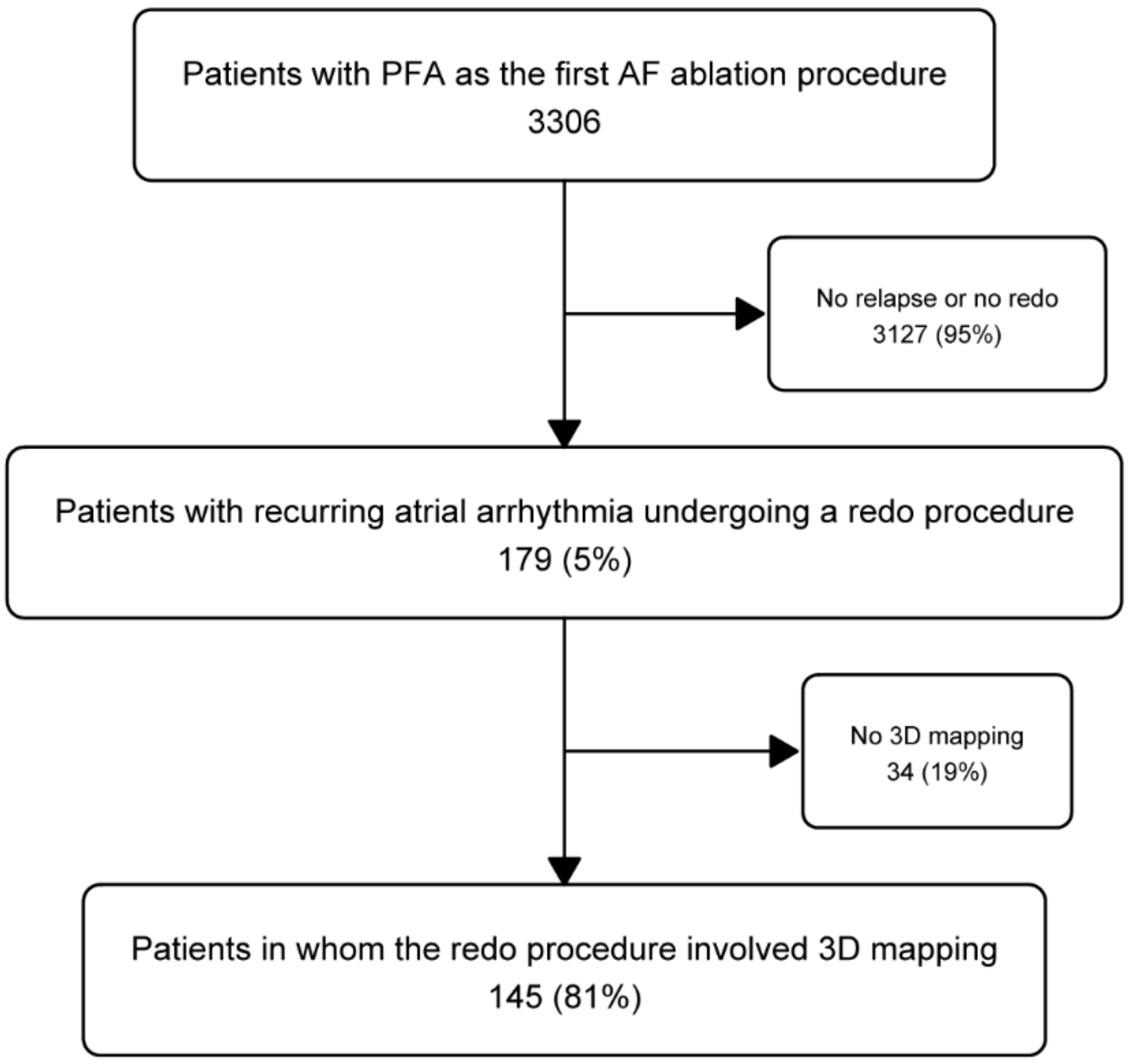

Supplemental Figure 2

Proportion of isolated PVs according to their location and the number of PFA applications they received

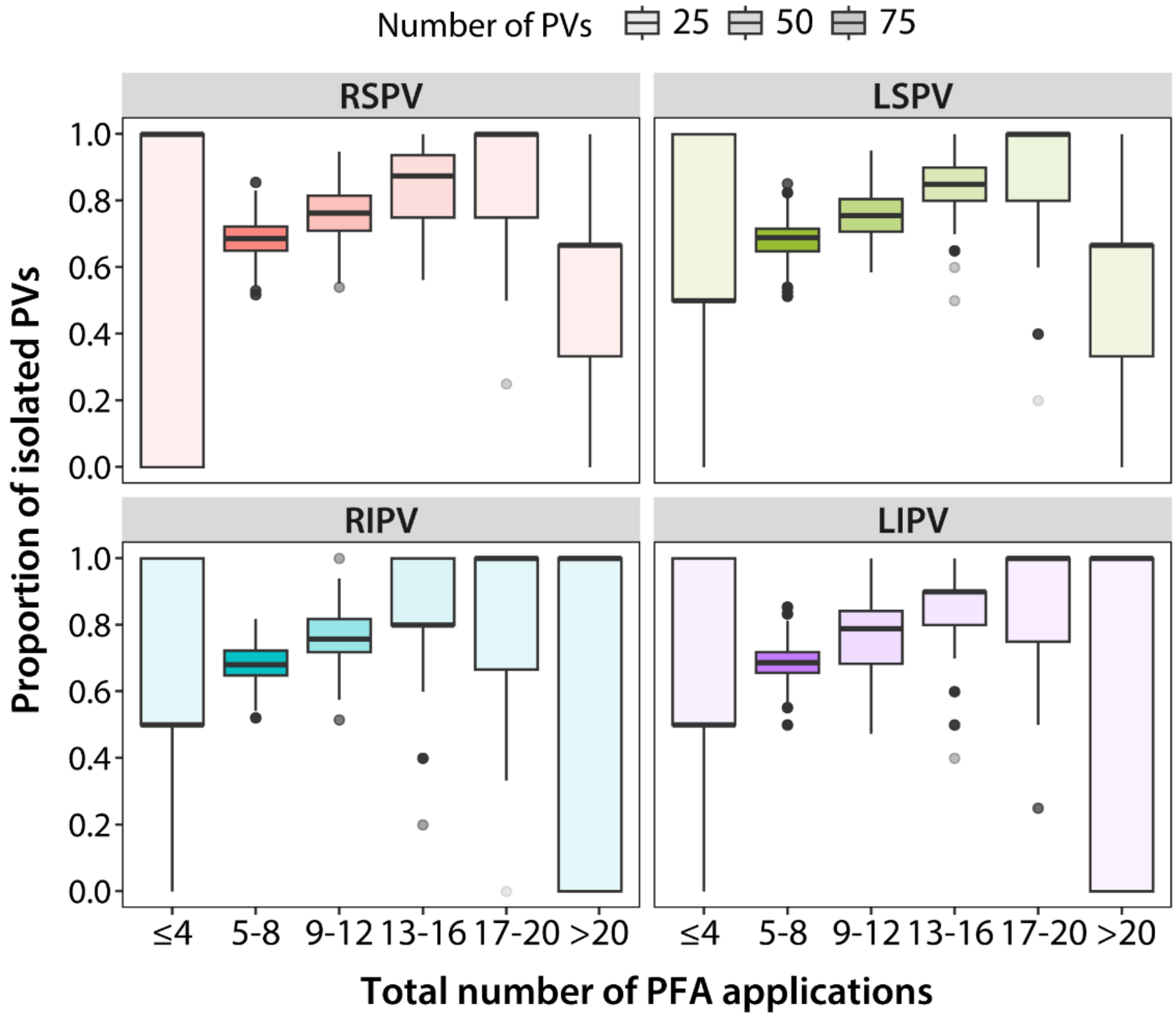

Line: proportion, boxplot boundaries: bootstrapped IQR, whisker boundaries: 1.5×bootstrapped IQR, dots: datapoints outside of whisker boundaries (outliers)
